# Surgical Interventions Following Radiotherapy in Spinal Metastases with Intermediate Instability: A Risk Factor Analysis: The Korean Society of Spinal Tumor Multicenter Study (KSST 2022-02)

**DOI:** 10.3390/cancers16142554

**Published:** 2024-07-16

**Authors:** Se-Jun Park, Jin Ho Kim, Yong Chan Ahn, Woong Sub Koom, Hwa Kyung Byun, Young-Hoon Kim, Sang-Il Kim, Dong-Ho Kang, on behalf of the Korean Society of Spinal Tumor

**Affiliations:** 1 Department of Orthopedic Surgery, Samsung Medical Center, Sungkyunkwan University, Seoul 06351, Republic of Korea; sejunos.park@samsung.com; 2 Department of Radiation Oncology, Seoul National University Hospital, Seoul National University, Seoul 03080, Republic of Korea; 3 Department of Radiation Oncology, Samsung Medical Center, Sungkyunkwan University, Seoul 06351, Republic of Korea; ahnyc@skku.edu; 4 Department of Radiation Oncology, Yonsei Cancer Center, Yonsei University, Seoul 03722, Republic of Korea; 5 Department of Radiation Oncology, Yongin Severance Hospital, College of Medicine, Yonsei University, Yongin 16995, Republic of Korea; hkbyun05@yuhs.ac; 6 Department of Orthopedic Surgery, Seoul St. Mary’s Hospital, Catholic University, Seoul 06591, Republic of Korea; boscoa@catholic.ac.kr (Y.-H.K.); luna2eternal@catholic.ac.kr (S.-I.K.)

**Keywords:** spinal metastasis, intermediate instability, risk factor, surgical intervention, radiotherapy

## Abstract

**Simple Summary:**

Spinal metastases can cause instability, making it difficult for clinicians to decide on the best treatment. While guidelines exist for stable and unstable spinal metastases, those with intermediate instability (a SINS of 7–12) pose a clinical challenge. This study examines risk factors that might require surgical intervention following initial radiotherapy in these patients. We analyzed data from 469 patients with spinal metastases treated between 2019 and 2021. Our findings show that patients with primary tumors in the lung, liver, or kidney, higher Bilsky grades of spinal cord compression, lytic bone lesions, and higher radiation doses are more likely to need surgery. These results suggest that the careful evaluation of these factors can help us to develop better treatment strategies for patients with intermediate spinal instability.

**Abstract:**

Background: One important determinant in choosing a treatment modality is spinal instability. Clear management guidelines are suggested for stable and unstable spinal metastatic lesions, but lesions in the intermediate instability category (SINS [spinal instability neoplastic score] score of 7–12) remain a clinical dilemma. This study aims to analyze the risk factors necessitating surgical intervention after radiotherapy (RT) in patients with those lesions. Methods: A multicenter cohort of 469 patients with spinal metastases of intermediate instability who received radiotherapy (RT) as the initial treatment between 2019 and 2021 were retrospectively enrolled. All patients were neurologically intact at the time of RT. According to the performance of surgical intervention after RT, various clinical and radiographic risk factors for surgical intervention were compared between surgery and non-surgery groups using uni- and multivariate analyses. A recursive partitioning analysis (RPA) was performed using significant determinants identified in multivariate analysis. Results: The mean age at the time of RT was 59.9 years and there were 198 females. The lung was the most common primary site. During the mean follow-up duration of 18.2 months, surgical treatment was required in 79 (17.9%) of patients. The most common surgical method was decompressive laminectomy with stabilization (62.0%), followed by vertebrectomy with stabilization (22.8%) and stabilization only (15.2%). The mean SINS for the total cohort was 9.0. Multivariate regression analyses revealed that the primary tumor site of the lung, liver, and kidney, higher Bilsky grades of ESCC, lytic bone lesions, and higher EQD2_10_ were significant risk factors for surgical intervention after RT. Among them, Bilsky grade, primary tumor type of the lung, liver, and kidney, and EQD2_10_ were the most important determinants for expecting the probability of surgical intervention on RPA. Conclusions: Surgical intervention was performed in 17.9% of patients with intermediate instability after RT as the initial treatment. The primary tumor site of the lung, liver, and kidney, higher Bilsky grade of ESCC, and EQD210 were the most important determinants for expecting the probability of surgical intervention. Therefore, the optimal treatment strategy needs to be devised by carefully evaluating the risk of surgical intervention.

## 1. Introduction

The spinal column is known as a common site of bone metastasis, and about 10% of all cancer patients have spinal metastases during the course of their disease [1,2]. Deciding the optimal treatment for patients with spinal metastases is frequently challenging because there are many clinical factors to consider. One important determinant in choosing a treatment modality is spinal instability [3]. The Spinal Oncology Study Group’s (SOSG) spinal instability neoplastic score (SINS) categorizes instability into stable, potentially unstable, and unstable: 0–6 points for stable, 7–12 for potentially unstable, and 13–18 for unstable [3,4]. While the guidelines for stable and unstable lesions are clear, managing potentially unstable lesions (SINS 7–12) remains uncertain as no defined recommendation exists regarding that category [3,5,6].

As this lesion is not considered overt instability, radiotherapy (RT) is frequently performed as the first-line treatment modality for patients in this category unless a patient suffers from progressive neurologic deficits [7,8]. In the last decade, vertebral compression fractures (VCFs) have gained attention as a common adverse event after RT [9,10,11,12,13,14,15,16,17]. The incidence of VCFs after RT is reported as high as 11.4–40.5% [9,11,13,14,15,16,17,18]. However, the clinical significance of VCFs remains controversial because not all VCFs increase pain or require salvage surgical intervention. In a recent systematic review by Faruqi et al., salvage surgical intervention was performed only in an average of 37% of patients for post-RT VCF [19]. Therefore, they recommended close observation before initiating surgical stabilization because the pain associated with the VCF may have settled.

Unlike radiographic VCF development, the need for surgical intervention after RT may be a more clinically relevant issue among both radiation oncologists and spine surgeons. For patients who are presumed to be at risk of necessitating surgical intervention, planned surgical treatment can be considered, or more meticulous follow-up is recommended even if the surgical treatment is not performed immediately. However, as evidenced so far, it appears to be difficult to determine which patients are at risk for surgical intervention, especially in patients with spinal metastases of the grey zone of instability. Therefore, the aim of this study was to analyze the risk factors necessitating surgical intervention after RT in spinal metastases with intermediate instability.

## 2. Materials and Methods

This retrospective multicenter study was approved by the local institutional review board and conducted in accordance with the ethical standards of the Declaration of Helsinki. 

### 2.1. Study Cohort

Four tertiary hospitals participated in this study. Consecutive patients with spinal metastases who underwent RT as the first treatment during 2019–2021 were initially screened. Among them, only patients with intermediate instability (SINS 7–12) who received RT as the first treatment were included in this study. All patients were neurologically intact at the time of RT. The SINS was retrospectively identified using pretreatment-available clinical information and planning CT and MRI imaging [3]. In cases of contiguous metastatic lesions within the treatment field, the lesion with the highest SINS was adopted. Additionally, ≥6-month follow-up after RT was required for inclusion. Patients were excluded if they underwent RT on more than two regions of the cervical, thoracic, lumbar, and sacrum region at the same time; needed urgent surgical treatment for progressive neurologic deficit at the time of RT; had previous RT at the same lesion; received planned prophylactic surgical stabilization before/after RT; were lost to follow-up or died within six months; or had incomplete radiographic examination or missing clinical data about pain nature for the SINS calculation. 

### 2.2. Outcome Measures

The primary endpoint of this study was the performance of the surgical intervention. The main indications for surgical treatment after RT were intractable pain caused by aggravating instability, neurologic deficit caused by spinal cord compression, or both. The decision to perform surgery was made by a spine tumor board in each institution. Multiple clinical factors, such as degree of pain, neurologic status, primary cancer type, performance status, response to previous RT, and systemic therapy sensitivity, were considered in decision-making. However, the presenting symptoms, general conditions, and primary tumor types were the most important considerations in determining the necessity of surgery as well as the surgical method. According to the performance of the surgical treatment after RT, two groups were created: group S (surgery group) and group NS (non-surgery group). Only patients who had surgical treatment under general anesthesia were included in group S. Therefore, nine patients who received vertebroplasty under local anesthesia were not considered as group S. However, four patients who were recommended for surgical treatment due to progressive neurologic deficit but did not receive it due to poor general condition or patient refusal were included in group S because their need for surgical treatment was agreed upon by the surgeons and patients. 

### 2.3. Data Collection

The dates of the last follow-up or death were collected. Data about the surgical interventions included the time from RT to surgery, the main causes of surgery (pain, neurologic deficit, and pain plus neurologic deficit), and the types of surgical treatment (stabilization only, decompressive laminectomy plus stabilization, and vertebrectomy plus stabilization). The vertebrectomy encompasses all procedures involving the removal of a metastasized vertebral body by either en-bloc or piecemeal manners. Patient demographics were obtained, including sex, age at the time of RT, primary tumor sites (top nine malignancies and others), and presence of visceral metastases (yes vs. no). A radiographic assessment of epidural spinal cord compression (ESCC) was conducted using Bilsky’s scale consisting of grades 0–3 on the MRI [20]. Grade 0 or 1 represents a disease confined to the bone or that with epidural space involvement but no spinal cord or cauda equina compression; grade 2 is an epidural impingement with spinal cord or cauda equina compression but a cerebrospinal fluid (CSF) signal is still present; grade 3 is spinal cord or cauda equina compression with the complete obliteration of a CSF signal. Six categories in the SINS system (location, pain, bone lesion, spinal alignment, vertebral body collapse, and posterolateral involvement) and their total sum were evaluated. In terms of treatment factors, the performance of systemic treatment and the use of antiresorptive agents, such as denosumab or bisphosphonate (yes vs. no), were assessed. Data about RT included the number of fractionations, radiation dose per one fraction (Gy), total radiation dose (Gy), equivalent dose in 2-Gy fractions_10_ (EQD2_10_), performance of stereotactic body radiation therapy (SBRT), and number of vertebral bodies included in the irradiation field.

### 2.4. Statistical Analysis

Data are presented as frequencies with percentages for the categorical variables and means with standard deviations for the continuous variables. The collected data were compared between the surgical intervention and non-surgical intervention groups. A Kaplan–Meier survivorship analysis was used to calculate the estimated survival time and time to surgical intervention after RT. A univariate analysis was performed using the Chi-square or Fisher’s exact tests to compare the categorical variables and independent t-tests or the Mann–Whitney U test to assess the differences in the means of the continuous variables between the two groups. A multivariate logistic regression analysis was performed using all variables with significance in the univariate analysis to identify the independent risk factors necessitating surgical intervention. Using the significant determinant on multivariate logistic regression analysis, recursive partitioning analysis (RPA) was performed to show the decision-making tree. Statistical analyses were conducted by professional statisticians using SPSS (version 27.0.0; IBM Corp., Armonk, NY, USA) and the rpart package of R program version 3.2.3 (R Foundation for Statistical Computing, Vienna, Austria). *p* < 0.05 was considered statistically significant.

## 3. Results

A total of 469 patients were identified to meet the inclusion criteria and constituted the study cohort. The mean age at the time of RT was 59.9 years, and there were 198 females and 271 males (Table 1). There were no significant differences between the two groups in terms of age and sex. With regard to the primary tumor, the lung was the most common site, followed by the liver, breast, kidney, colorectum, prostate, sarcoma, nasopharynx, pancreas, and others. The proportion of specific primary tumor origins between the two groups was significantly different (*p* = 0.022). The rate of surgical treatment was the highest in patients with kidney tumors (11 out of 32, 34.4%) and the lowest in patients with breast tumors (3 out of 63, 4.8%). Nearly half of the patients had visceral metastases, but there was no significant difference between the two groups with regard to the presence of visceral metastases. In terms of the Bilsky grade of ESCC, there were 113 patients (24.7%) with grade 0, 206 (45.1%) with grade 1, 105 (23.0%) with grade 2, and 33 (7.2%) with grade 3 before RT. The Bilsky grades significantly differed between the two groups (*p* < 0.001). Surgical treatment was performed in 4.4% (5 out of 113) of patients with grade 0, 14.1% (29 out of 206) with grade 1, 21.0% (22 out of 105) with grade 2, and 42.4% (14 out of 33) with grade 3.

**Figure 1:**
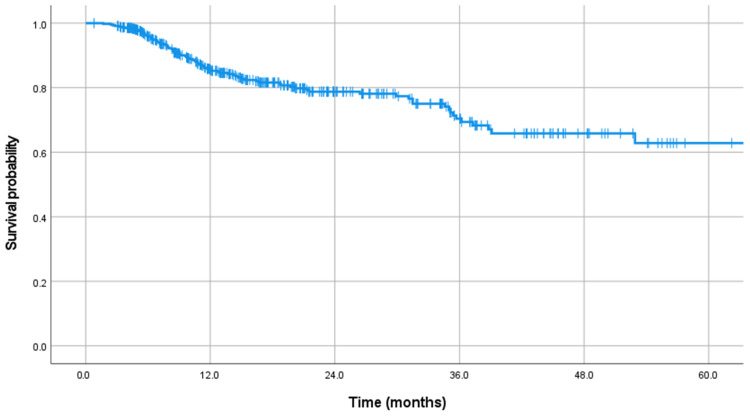
Kaplan–Meier curve showing the survival time.

**Figure 2:**
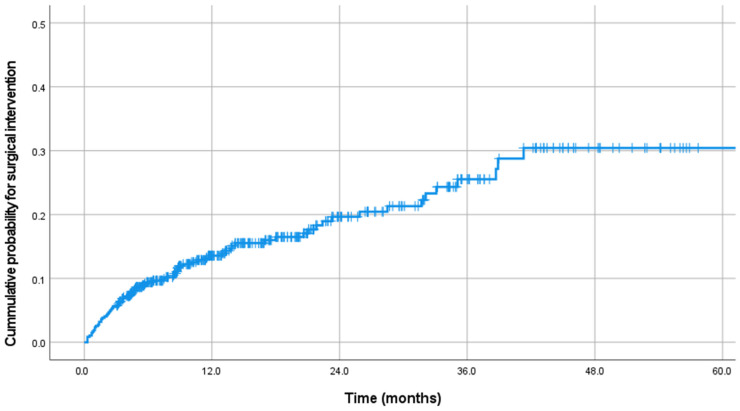
Kaplan–Meier curve showing the cumulative probability of surgical intervention.

After RT, the mean follow-up duration was 18.2 months (Table 2). The estimated median survival time was 43.6 months (Figure 1). An additional Kaplan–Meier analysis to compare the survival between group S and group NS showed that the survival in group S was worse than in group NS (*p* < 0.001, Appendix A). After RT, 310 patients did not require further local treatment. However, re-RT was performed in seventy-one patients, vertebroplasty was performed in nine, surgery was performed in seventy-five, and surgery was recommended for four patients. Therefore, 79 patients (17.9%) who received surgical intervention or were recommended for surgery were included in group S. Surgical intervention was performed after a mean of 10.3 months (Figure 2). Most surgical treatments were performed within one year after RT, with a surgical rate of 72.2% within one year, 15.1% between one and two years, and 12.7% after two years. The main symptoms for surgery were pain in 30 patients (40.0%), neurologic deficit in 17 (21.5%), and combined pain and neurologic deficit in 32 patients (40.5%). The primary surgical treatment method was decompressive laminectomy with stabilization (62.0%), followed by vertebrectomy with stabilization (22.8%) and stabilization only (15.2%). In the vertebrectomy group, only two patients had total en-bloc spondylectomy, while twelve underwent piecemeal vertebrectomy through a posterior approach.

The mean total SINS was 9.0 for the entire cohort, but there was no difference between the two groups (Table 3). The patient distribution in each SINS category is listed in Table 3. With regard to the SINS categories, pain, bone lesion, and vertebral body collapse significantly differed between the two groups, while location, spinal alignment, and posterolateral involvement did not. Patients with mechanical pain underwent surgical treatment in 25.0% (31 out of 124) of the population, which was a higher rate compared to patients with occasional pain (45 out of 330, 13.6%) or patients without pain (3 out of 15, 20%). With regard to the bone lesions, the rate of surgical intervention was highest for lytic lesions (64 out of 322, 19.9%), followed by mixed (14 out of 130, 10.8%) and blastic lesions (1 out of 17, 5.9%). In terms of vertebral body collapse, surgical treatment was performed most frequently in patients with VCF with >50% collapse (13 out of 39, 33.3%), followed by patients with VCF < 50% collapse (25 out of 158, 15.8%), patients with >50% body involvement (27 out of 164, 16.5%), and patients without VCF or >50% body involvement (14 out of 106, 13.2%). 

Patients underwent systemic treatment in 65.2% of cases and received an antiresorptive agent in 8.3% (Table 4). However, there were no significant differences in terms of systemic treatment or use of antiresorptive agents between the two groups. The mean number of fractionations was 5.0, and it did not differ between the groups. SBRT was used in 179 patients (38.2%). There was no difference in SBRT performance between the two groups. The mean fraction dose and total radiation dose were also not different between the two groups. However, EQD2_10_ was significantly greater in group S than in group NS (34.7 Gy vs. 30.5 Gy, *p* = 0.049). The number of vertebral bodies in the irradiation field was one in 42.2% of patients, two in 14.5%, and three or more in 43.3%; there was no difference in this parameter between the two groups.

**Figure 3:**
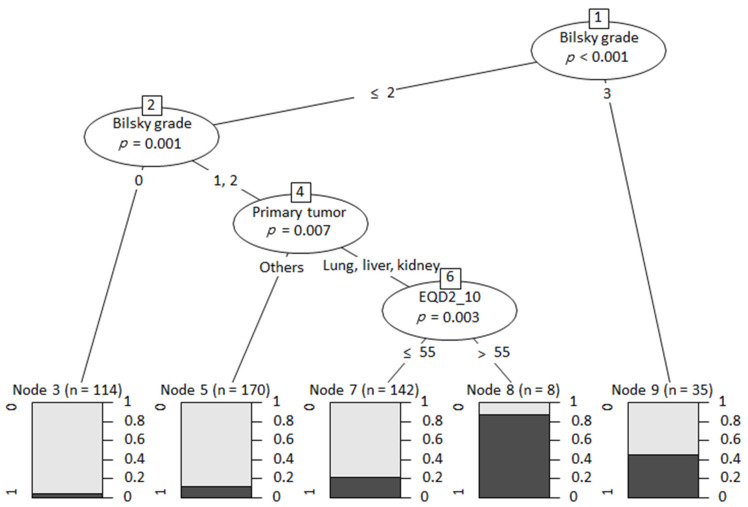
Decision tree using recursive partitioning analysis.

The multivariate logistic regression analysis revealed that the primary tumor site of the lung, liver, and kidney, a higher Bilsky grade for ESCC, lytic bone lesions, and higher EQD2_10_ values were significant risk factors for surgical intervention after RT (Table 5). In terms of the primary tumor site, patients with metastases from the lung, liver, and kidney were significantly more likely to require surgical treatment compared to others as a reference (odds ratio [OR] = 2.871 for lung, OR = 2.891 for liver, and OR = 5.588 for kidney). The Bilsky grade for ESCC showed a strong positive correlation with the odds of surgical treatment (OR = 3.602 for grade 1, OR = 6.374 for grade 2, and OR = 19.966 for grade 3). Lytic bone lesions increased the risk for surgical intervention by 2.175 times compared with blastic lesions. Higher EQD2_10_ values minimally increased the odds of surgical intervention (OR = 1.014). Among these significant risk factors, RPA showed that three components, Bilsky grade, primary tumor type, and EQD2_10_, were significant determinants for expecting the probability of surgical intervention (Figure 3).

## 4. Discussion

Mechanical instability is one of the main indications requiring surgical intervention in patients with spinal metastases [21,22,23,24]. The SINS system, validated through numerous studies, categorizes lesions into stable, intermediate stability, and instability [9,11,13,14,15,16,17,18,25,26]. Standard recommendations advise observation for stable (SINS 0–6) and prophylactic stabilization for unstable lesions (SINS 13–18). The NOMS (neurologic, oncologic, mechanical, and systemic) framework, a widely used treatment strategy, also classifies lesions into stable and unstable but lacks guidance for intermediate instability (SINS 7–12) [8]. Our multicenter study addresses this gap by identifying the risk factors for surgical intervention after RT in this grey zone.

New VCF development and the progression of existing VCFs are common adverse events after RT. During the past decade, VCF development has been a major concern after RT and was used as a primary endpoint of numerous risk factor studies [19,25,26]. However, not all VCFs are clinically problematic. Shi et al. assessed VCF development after conventional RT and found that twenty-one (41.2%) of fifty-one VCF lesions were symptomatic, but only two lesions (3.9%) required open invasive surgery [13]. According to a recent long-term prospective phase two study by Mantel et al., post-SBRT VCF developed in 21 of 61 lesions (34.4%) [10]. However, only three of the twenty-one VCFs were associated with a pain increase of two or more on the VAS, and only three (3.6%) of the total study cohort (fifty-one patients) had to be treated surgically due to a VCF. Therefore, we thought that it was necessary to perform a study with a primary endpoint of surgical intervention in order to add up the clinical relevance. Salvage surgical intervention is typically indicated in patients with intractable pain caused by a VCF or a progressive neurologic deficit caused by ESCC. However, it is sometimes difficult to clearly differentiate the exact cause for surgical treatment between instability and ESCC because many lesions necessitating surgical treatment develop with a combination of pain and neurologic deficit. The current study showed that patients with mixed pain and neurologic deficits accounted for 40.5% of total surgical cases. Although the patients presented mainly with neurologic deficits, ESCC occurrence indicated the breakdown of the posterior cortical integrity of the vertebrae, which was already associated with spinal instability to some extent. In the current study, we also found that a stabilization-only procedure was performed in only 15.2% of patients, while pain as the main cause of surgery accounted for 40.0%. 

In this study, we found that the primary tumor site significantly affected the risk for salvage surgical intervention. Compared to other primary tumor sites, metastases from the lung, liver, and colorectum showed a significantly higher risk for surgical treatment. The effect of primary tumor type on the probability of further surgical treatment can be explained by radiosensitivity. It is well-known that radiosensitivity varies according to the primary tumor. If the metastatic lesion shows a good response to RT in terms of pain reduction and neurologic improvement, the probability of further surgical treatment is expected to be reduced. There are a few previous reports that are consistent with our results. Bernard et al. evaluated the local control rate after stereotactic radiosurgery in patients with spinal metastases traditionally known as radiosensitive tumors, such as lung, colon, thyroid, and breast cancers [27]. Among these four tumor types, they found that spinal metastases from lung cancers showed a propensity toward local failure compared to other primary tumor types. Shi et al. also found that lung cancer had a 1.91-fold higher risk for new or worsening VCF after conventional RT compared to breast and prostate cancer [13]. Cunha et al. also found that a higher risk of VCFs after SBRT was found in patients with lung and hepatocellular tumors [18]. Renal cell carcinoma is also known as a relatively radioresistant tumor, causing osteolytic metastatic lesions and frequently necessitating surgical treatment [15,16]. However, caution must be applied to interpret the results regarding the association of primary tumor types and the risk of further salvage surgical treatment. First, radiosensitivity may differ between the primary cancer site and metastatic lesions because it is possible that metastatic disease may confer a more radioresistant phenotype compared with those treated at the primary site [27]. In addition, the failure rate in local metastatic lesions can also be affected by the responsiveness to systemic medical treatment or the use of an antiresorptive agent. Lastly, the biological behavior, even in the same primary tumor type, can vary according to subtype and gene mutation status. Therefore, the effect of the primary tumor site on failure after RT demonstrated in the previous studies and our study could provide rough guidance to physicians, but detailed clinical situations should be taken into consideration together with assessing the radiosensitivity of a certain cancer.

The Bilsky grade for ESCC is a crucial parameter for evaluating the probability of surgical intervention because a neurologic deficit is the main cause for requiring surgical treatment. In the current study, a higher Bilsky grade at the time of RT was strongly associated with a higher risk of surgical treatment. The SINS system can provide the severity of mechanical instability only in the aspect of the bony structure. However, any metastatic lesions cannot be evaluated solely within a bony structure because neurologic components are also important factors to consider when selecting a treatment modality. Therefore, we believe that the ESCC grade should always be evaluated together with mechanical instability in assessing the durability of metastatic lesions.

The SINS system can be usefully applied to differentiate stable and unstable lesions to predict VCF development or salvage surgical intervention after RT. Aiba et al. reported outcomes in 47 patients with spinal metastases from lung cancer [14]. After categorizing patients into stable (SINS 1–6) and unstable (SINS 7–18) groups, they found that patients with unstable lesions were nearly four-fold more likely than those with stable lesions to experience a skeletal-related event. Versteeg et al. compared SINSs between operative and non-operative cases in a multicenter series of 1509 patients. They also found significantly higher SINSs in the operative group compared with the non-operative group (10.7 vs. 7.2 points) [28]. However, in the current study, we did not find a difference in total SINSs between the surgery and non-surgery groups. We feel the total SINS is useful in discriminating between stable and unstable lesions in the general population with spinal metastases, but it may not be accurate in distinguishing those two lesions within this intermediate instability category. Instead, we found three categories of the SINS system, pain, bone lesion, and collapse, significantly affected the odds of surgical treatment in our univariate analysis. Our finding is consistent with the results of previous studies reporting that these three components significantly increase the odds of new VCF development or worsening VCFs [9,13,15,17,18]. However, multivariate analysis in the current study revealed that only lytic bone lesions were a significant risk factor among six SINS categories and increased the odds of surgical treatment by 2.1-fold compared to blastic and mixed lesions.

EQD2_10_ is the equivalent radiation dose of a radiotherapy plan in standard 2 Gy fractions. It helps evaluate the effectiveness and toxicity of radiation therapy, especially when comparing various fractionation methods. Our study found that EQD2_10_ was significantly greater in group S than in group NS (34.7 Gy vs. 30.5 Gy, *p* = 0.049). Furthermore, the multivariate logistic regression analysis revealed that higher EQD2_10_ values were a significant risk factor for surgical intervention after radiation therapy. This result is echoed by Kim’s study, which showed that the larger the area that receives high-dose RT, the greater the probability of vertebral compression fracture [12]. This indicates that patients in the surgical group received a higher biologically effective dose of radiation therapy compared to the non-surgical group, and a higher EQD2_10_ could be associated with an increased likelihood of requiring surgical intervention following radiation therapy. These findings imply that patients with spinal metastasis receiving RT of higher EQD210 need close follow-up for early detection and the necessity of surgical intervention in patients undergoing radiation therapy for spinal metastases. Incorporating EQD210 into clinical practice may help in stratifying patients based on their risk and in making more informed decisions regarding their treatment plans. Further research is warranted to validate these findings and to explore the potential of EQD210 as a predictive marker in larger, multicenter cohorts.

Our study did not determine whether surgery for patients in the intermediate instability group is more beneficial to survival compared to no surgery. An additional Kaplan–Meier analysis to compare the survival between group S and group NS showed that the survival in group S was worse than in group NS (*p* < 0.001, Appendix A). This finding suggests that various factors, such as the worse preoperative functional status in group S, might contribute to the observed differences in survival outcomes. To accurately compare the effect of different treatments on survival, additional studies are required where preoperative oncologic factors and functional status are matched between the surgical and non-surgical groups, allowing for a comparison of treatment methods within comparable cohorts. 

A few limitations should be acknowledged in this study. First, although we performed a multicenter study including a large number of patients, the retrospective nature of the current study is an inherent limitation. Second, the multicenter nature of the study introduced variability in the treatment protocols, surgical decision-making, and follow-up practices across different institutions. The decision about the necessity, timing, and method of surgery could have been subjective and varied significantly between individual centers and surgeons. This heterogeneity may impact the generalizability of our findings. We also think that decisions of technique, dose, and timing of RT might have the same potential bias as surgical treatment. Third, lesions with Bilsky grade 3 (7.2%) were included in this study, and they might not be a good indication for RT because a sufficient radiation dose cannot be delivered to metastatic lesions due to potential neural toxicity [29,30]. However, if the patient does not present with a neurologic deficit, even in radiographic Bilsky grade 3 lesions, surgical treatment is frequently waived, and RT can be a preferred option [31,32]. Lastly, our study did not assess the data on the severity of neurological deficits, such as the Frankel grade, that occurred after irradiation. However, we confirmed that paralysis was a key symptom influencing the decision for surgery, with most patients likely having Frankel grade 3 or 4. Additionally, the results include Bilsky grade information, indicating the extent of cord compression and providing indirect information about functional status. Understanding the natural history of neurological decline after irradiation for these patients may provide useful information to surgeons in clinical settings. Therefore, further research should include this information and derive results.

Despite some critical limitations, this study could suggest a treatment strategy for this patient group of intermediate instability. In patients with a high risk for surgical intervention after RT, planned stabilization should be considered in cases with a low grade of ESCC, and decompression plus stabilization can be considered in cases with a high grade of ESCC. Even if the surgical treatment is not performed immediately after RT for these high-risk patients, close observation is recommended with a short follow-up duration.

## 5. Conclusions

Surgical intervention was performed in 17.9% of patients with intermediate instability after RT as the initial treatment. The multivariate analysis showed that the risk factors for surgical treatment were the primary tumor site of the lung, liver, and kidney, a higher Bilsky grade of ESCC, lytic bone lesions, and higher radiation doses. Among them, the Bilsky grade, primary tumor type, and EQD2_10_ were the most important determinants for expecting the probability of surgical intervention. Therefore, the optimal treatment strategy needs to be devised by carefully evaluating the risk of surgical intervention.

## Figures and Tables

**Table 1:** Baseline patient and tumor characteristics and their comparison between the two groups.

Parameter	Total	Group S	Group NS	*p* Value
Age, years	59.9 ± 12.7	58.5 ± 9.9	60.2 ± 13.1	0.309
Male, *n* (%)	271 (57.8%)	51 (64.6%)	220 (56.4%)	0.212
Primary tumor site, *n* (%)				**0.022**
Lung, *n* (%)	123 (26.2%)	25 (31.6%)	98 (25.1%)	
Liver, *n* (%)	72 (15.4%)	15 (19.0%)	57 (14.6%)	
Breast, *n* (%)	63 (13.4%)	3 (3.8%)	60 (15.4%)	
Kidney, *n* (%)	32 (6.8%)	11 (13.9%)	21 (5.4%)	
Colorectum, *n* (%)	27 (5.8%)	5 (6.3%)	22 (5.6%)	
Prostate, *n* (%)	21 (4.6%)	2 (2.5%)	19 (4.9%)	
Sarcoma, *n* (%)	21 (4.6%)	3 (3.8%)	18 (4.6%)	
Nasopharynx, *n* (%)	11 (2.3%)	3 (3.8%)	8 (2.1%)	
Pancreas, *n* (%)	10 (2.2%)	2 (2.5%)	8 (2.1%)	
Others, *n* (%)	89 (19.0%)	10 (12.7%)	79 (20.3%)	
Presence of visceral metastases				0.796
Yes, *n* (%)	221 (48.4%)	35 (50.0%)	186 (48.1%)	
No, *n* (%)	236 (51.6%)	35 (50.0%)	201 (51.9%)	
Bilsky grade for ESCC				**<0.001**
Grade 0, *n* (%)	113 (24.7%)	5 (7.1%)	108 (27.9%)	
Grade 1, *n* (%)	206 (45.1%)	29 (41.4%)	177 (45.7%)	
Grade 2, *n* (%)	105 (23.0%)	22 (31.4%)	83 (21.4%)	
Grade 3, *n* (%)	33 (7.2%)	14 (20.0%)	19 (4.9%)	

Bold *p* values mean statistical significance. ESCC: epidural spinal cord compression.

**Table 2:** Follow-up data after radiotherapy.

Parameter	Statistics
Follow-up duration, months	18.2 ± 13.4
Survival data	*n* = 469
Number of alive patients, *n* (%)	379 (80.8%)
Estimated median survival time after radiotherapy (months)	43.6 ± 1.3
Further treatment after radiotherapy	*n* = 469
No further local treatment, *n* (%)	310 (66.1%)
Re-radiotherapy, *n* (%)	71 (15.1%)
Vertebroplasty, *n* (%)	9 (1.9%)
Surgery, *n* (%)	75 (16.0%)
Surgery recommended but not performed, *n* (%)	4 (0.9%)
Surgical intervention	*n* = 79
Mean time from radiotherapy, months	10.3 ± 12.1
Main symptoms of surgical intervention	
Pain, *n* (%)	30 (40.0%)
Neurologic deficit, *n* (%)	17 (21.5%)
Pain and neurologic deficit, *n* (%)	32 (40.5%)
Types of surgical treatment	
Stabilization only, *n* (%)	12 (15.2%)
Decompressive laminectomy with stabilization, *n* (%)	49 (62.0%)
Vertebrectomy with stabilization, *n* (%)	18 (22.8%)

**Table 3:** Distribution of SINS categories and their comparisons between the two groups.

SINS Category	Total	Group S	Group NS	*p* Value
Total sum of SINS, mean ± SD	9.0 ± 1.5	9.1 ± 1.7	8.9 ± 1.5	0.430
Location				0.794
Junctional, *n* (%)	235 (50.1%)	38 (48.1%)	197 (50.5%)	
Mobile, *n* (%)	135 (28.8%)	22 (27.8%)	113 (29.0%)	
Semi-rigid, *n* (%)	97 (20.7%)	19 (24.1%)	78 (20.0%)	
Rigid, *n* (%)	2 (0.4%)	0 (0%)	2 (0.5%)	
Pain				**0.015**
Yes, *n* (%)	124 (26.4%)	31 (39.2%)	93 (23.8%)	
Occasional pain but not mechanical, *n* (%)	330 (70.4%)	45 (57.0%)	285 (73.1%)	
Pain-free lesion, *n* (%)	15 (3.2%)	3 (3.8%)	12 (3.1%)	
Bone lesion				**0.030**
Lytic, *n* (%)	322 (68.7%)	64 (81.0%)	258 (66.2%)	
Mixed (lytic/blastic), *n* (%)	130 (27.7%)	14 (17.7%)	116 (29.7%)	
Blastic, *n* (%)	17 (3.6%)	1 (1.3%)	16 (4.1%)	
Spinal alignment				0.367
Subluxation/translation present, *n* (%)	1 (0.2%)	0 (0%)	1 (0.3%)	
De novo deformity, *n* (%)	68 (14.5%)	7 (8.9%)	61 (15.6%)	
Normal alignment, *n* (%)	400 (85.3%)	72 (91.1%)	328 (84.1%)	
Vertebral body collapse				**0.034**
>50% collapse, *n* (%)	39 (8.4%)	13 (16.5%)	26 (6.7%)	
<50% collapse, *n* (%)	158 (33.8%)	25 (31.6%)	133 (34.3%)	
No collapse with >50% body involved, *n* (%)	164 (35.1%)	27 (34.2%)	137 (35.3%)	
None of the above, *n* (%)	106 (22.7%)	14 (17.7%)	92 (23.7%)	
Posterolateral involvement				0.143
Bilateral, *n* (%)	90 (19.2%)	21 (26.6%)	69 (17.7%)	
Unilateral, *n* (%)	238 (50.7%)	39 (49.4%)	199 (51.0%)	
None of the above, *n* (%)	141 (30.1%)	19 (24.1%)	122 (31.3%)	

Bold *p* values mean statistical significance. SINS: spinal instability neoplastic score.

**Table 4:** Distribution of treatment factors and their comparisons between the two groups.

Parameter	Total	Group S	Group NS	*p* Value
Under systemic treatment				0.796
Yes, *n* (%)	306 (65.2%)	53 (17.3%)	26 (16.0%)	
No, *n* (%)	163 (34.8%)	253 (82.7%)	137 (84.0%)	
Use of antiresorptive agent				0.123
Yes, *n* (%)	39 (8.3%)	3 (7.7%)	76 (17.7%)	
No, *n* (%)	430 (91.7%)	36 (92.3%)	354 (82.3%)	
Number of fractionations	5.0 ± 3.7	4.9 ± 3.7	5.4 ± 3.6	0.303
SBRT				1.000
Yes, *n* (%)	179 (38.2%)	30 (38.0%)	149 (38.2%)	
No, *n* (%)	290 (61.8%)	49 (62.0%)	241 (61.8%)	
Radiation dose per fraction, Gy	8.2 ± 6.5	7.8 ± 6.5	8.2 ± 6.5	0.601
Total dose, Gy	23.8 ± 7.5	25.5 ± 10.3	23.4 ± 6.6	0.054
EQD2_10_, Gy	31.2 ± 17.3	34.7 ± 20.1	30.5 ± 16.6	**0.049**
Number of vertebral bodies irradiated				0.082
1, *n* (%)	198 (42.2%)	37 (46.8%)	161 (41.3%)	
2, *n* (%)	68 (14.5%)	16 (20.3%)	52 (13.3%)	
≥3, *n* (%)	203 (43.3%)	26 (32.9%)	177 (45.4%)	

Bold *p* values mean statistical significance. SBRT: stereotactic body radiation therapy; EQE2_10_: equivalent dose in 2-Gy fractions_10_.

**Table 5:** Multivariate logistic regression analysis of risk factors for surgical intervention.

Variable	OR (95% CI)	*p* Value
Primary tumor site		**0.043**
Others	Reference	
Lung	2.871 (1.138–6.441)	**0.023**
Liver	2.891 (1.127–6.512)	**0.034**
Breast	0.542 (0.135–2.174)	0.387
Kidney	5.588 (1.731–14.919)	**0.003**
Colorectum	1.797 (0.491–6.582)	0.376
Prostate	1.007 (0.195–5.752)	0.994
Sarcoma	1.033 (0.285–5.330)	0.806
Nasopharynx	3.212 (0.708–16.015)	0.145
Pancreas	3.568 (0.497–19.314)	0.176
Bilsky grade for ESCC		**<0.001**
Grade 0	Reference	
Grade 1	3.602 (1.299–9.650)	**0.014**
Grade 2	6.374 (2.144–17.600)	**0.001**
Grade 3	19.966 (5.662–60.038)	**<0.001**
SINS pain		0.119
No pain	Reference	
Occasional pain	2.156 (0.486–9.558)	0.312
Mechanical pain	1.200 (0.278–5.170)	0.807
SINS bone lesion		0.068
Blastic	Reference	
Mixed	1.957 (0.216–17.745)	0.210
Lytic	2.175 (1.117–4.143)	**0.020**
SINS-ollapse		0.613
No collapse	Reference	
Body involvement >50%	1.142 (0.256–2.480)	
Collapse <50%	0.950 (0.432–2.088)	
Collapse >50%	1.749 (0.640–4.781)	
EQD2_10_	1.014 (1.001–1.028)	**0.046**

Bold *p* values mean statistical significance. ESCC: epidural spinal cord compression; EQE2_10_: equivalent dose in 2-Gy fractions_10_.

**Figure A1:**
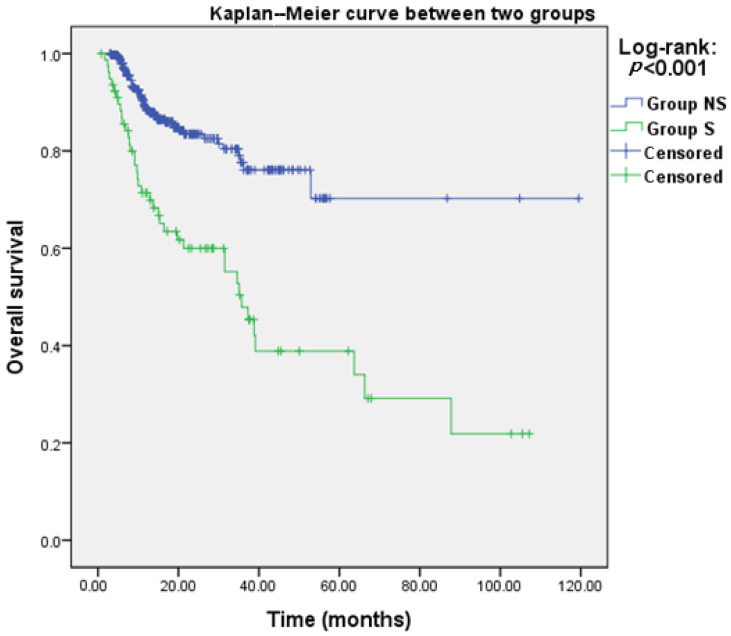
Kaplan–Meier curve of group S and group NS.

## Data Availability

Data underlying this article cannot be shared publicly due to the privacy of the individuals who participated in this study. The data may be shared upon a reasonable request by the corresponding author.
